# Supplementary material for: Using simulation to teach nursing students how to deal with a euthanasia request
Source: PLoS One. 2024 Mar 28;19(3):e0299049. doi: 10.1371/journal.pone.0299049 (PMC10977752; doi:10.1371/journal.pone.0299049)
Supplement: S1 Appendix — (DOCX) [file pone.0299049.s001.docx]

**Focus groups - dealing with end of life**

**Moderator:** ...................................................

**Observer:** ......................................................

Good evening, everyone. First and foremost, I would like to thank you all on behalf of the entire research team for being here. Your input is invaluable to us in gaining a better understanding of your experiences and optimizing the "dealing with end of life" module based on your feedback.

**Opening**

Could you please tell us your name, field of study, and the reason for your participation in these simulation trainings (possibly your interest in the topic)?

**Introduction**

Have you already had simulation trainings? If so, how was this simulation training different from previous ones?

**Transition**

What were your general experiences with this simulation training?

**Main Questions**

How did you feel about being involved in a simulation? What thoughts came to mind? How comfortable did you feel? What impact did this have on your confidence as a future caregiver? (pre-simulation)

What was it like to prepare for the simulation? What thoughts came to mind? How comfortable did you feel? What impact did this have on your confidence as a future caregiver? (briefing)

What was it like to be in a simulation? What is your perception of the realism level of the scenario? How comfortable did you feel? What impact did this have on your confidence as a future caregiver? (scenario)

How did the debriefing process feel to you? What thoughts came to mind? How comfortable did you feel? What impact did this have on your confidence as a future caregiver? (post-simulation discussion)

How did it feel to be recorded on video? What were your experiences with watching the video? What did you feel when you saw your performance? How comfortable did you feel? What impact did this have on your confidence as a future caregiver? (debriefing)

How did you feel immediately after the simulation? How long did those feelings last? Have your perceptions of simulation training changed since then? What impact does this have on your functioning as a future caregiver? (post-simulation)

Were there any factors that positively influenced your performance today?

Were there any factors that hindered your performance today?

Do you have any suggestions to improve these simulation trainings?

**Closing**

Is there anything else you would like to share with us regarding these simulation trainings?

**Probes** (additional questions to avoid misinterpretations and keep the conversation going)

- Can you tell us more about that?
- To better understand, what do you actually mean by that?
- Could you provide an example of that?
- Could you give another example of that?
