# Supplementary material for: Using simulation to teach nursing students how to deal with a euthanasia request
Source: PLoS One. 2024 Mar 28;19(3):e0299049. doi: 10.1371/journal.pone.0299049 (PMC10977752; doi:10.1371/journal.pone.0299049)
Supplement: Attachment — Submitted filename: Response to Reviewers.docx [file pone.0299049.s002.docx]

**REBUTTAL LETTER – RESPONSE TO THE EDITOR AND REVIEWERS**

**Manuscript ID PONE-D-23-21618**

**Using simulation to teach nursing students how to deal with a euthanasia request**

| **COMMENTS OF THE EDITOR** | **RESPONSE OF AUTHORS** |
| --- | --- |
|  | |
| **EDITORS’ COMMENTS** | |
|  | |
| Please ensure that your manuscript meets PLOS ONE's style requirements, including those for file naming. | We believe we are now in compliance with the journal's guidelines. |
| We note that the grant information you provided in the ‘Funding Information’ and ‘Financial Disclosure’ sections do not match.  When you resubmit, please ensure that you provide the correct grant numbers for the awards you received for your study in the ‘Funding Information’ section. | We have paid the necessary attention to this while resubmitting. |
| In your Data Availability statement, you have not specified where the minimal data set underlying the results described in your manuscript can be found. PLOS defines a study's minimal data set as the underlying data used to reach the conclusions drawn in the manuscript and any additional data required to replicate the reported study findings in their entirety. All PLOS journals require that the minimal data set be made fully available. | We have anonymized the three transcribed discussion groups and uploaded them to Mendeley. |

| **REVIEWER 1** | | |
| --- | --- | --- |
| **FORMAT** | | |
| Please correct the in-text citations. Citations need to be included in the sentence (i.e., citations with brackets before punctuation), and blank spaces are needed between characters and brackets. | We positioned all citations prior to the endpoint. | |
| **TITLE** | | |
| Suggest mentioning the “students’ perceptions towards simulation” in the title since it is a key aspect of the results. | We thank the reviewer for this suggestion. We changed the title. | |
| Using simulation to teach nursing students how to deal with a euthanasia request  Nursing students’ perceptions towards simulation on euthanasia | | |
| **ABSTRACT** | | |
| Suggest adding more details about the methods, such as participants and data analysis. | | We have revised the abstract based on your feedback. |
| In the results section, please add a sentence to describe the sample characteristics. | | We have revised the abstract based on your feedback. |
| Please make sure that the abstract does not exceed 300 words. | | The revised abstract now consists of 299 words. |
| Some sentences are a bit confusing, such as “Some students find it a useful tool, others find it too guiding.” and “However, specific nuances should be provided and therefore the module adapted to varied geographical contexts.” Please express these idea succinctly and clearly. | | We have revised the abstract based on your feedback and deleted these sentences. |
| Nursing students are confronted with euthanasia during their internship and certainly during their later career but they feel inadequately prepared in dealing with a euthanasia request. This study presents a simulation module focused on euthanasia and evaluates nursing students' perceptions after they have completed the simulation practice. The ‘euthanasia module’ consisted of a preparatory online learning module, a good-practice video, an in-vivo simulation scenario, and a debriefing session. The module’s content was validated by four experts in end-of-life care. The module was completed by three groups of students from two different University Colleges (n=17 in total). The students were divided into three groups: one with no previous simulation education experience, one with all students having previous experience, and another with a mix of experiences. After completing the entire module, each group had a discussion regarding their perceptions and expectations concerning this simulation module. Thematic content analysis was conducted on audio-recorded group interviews using NVIVO® software, involving initial open coding, transformation into specific themes and subthemes through axial coding, and defining core themes through selective coding, with data analysis continuing until data saturation was reached. Students generally found the online learning module valuable for background information, had mixed perceptions of the good-practice video, and appreciated the well-crafted scenarios with the taboo of euthanasia emerging during simulations, while the debriefing process was seen as enhancing clinical reasoning abilities. Students considered the simulation module a valuable addition to their education and nursing careers, expressing their satisfaction with the comprehensive coverage of the sensitive topic presented without sensationalism or taboos. This subject holds significance for nations with established euthanasia laws and those lacking such legal frameworks. The findings of this study can aid teachers in developing and accessing euthanasia simulation training programs, contributing to broader education's emphasis on integrating euthanasia-related knowledge and skills. | | |
| **INTRODUCTION** | | |
| The author provides much information on simulation that does not show a close association with the research question. Please revise the introduction and demonstrate the dilemmas regarding euthanasia encountered by nursing students thoroughly to make the gap clear and the research rationale sound. | | We have made efforts to clarify the dilemmas and condense the section on simulation. |
| From lines 58 to 59, the author provides the definition of physician-assisted suicide which is not closely associated with the research question. | | We deleted this sentence. |
| In lines 66 and lines 73, the author emphasizes unbearable mental suffering (UMS-euthanasia), yet the intervention in this study is not solely for UMS-euthanasia. | | This is a valid observation; however, we could not locate any studies that investigate students' knowledge regarding euthanasia in general, but only specifically pertaining to UMS-euthanasia. We have added an additional sentence to clarify this. |
| In Belgium, handling euthanasia requests follows a rigorous procedure, with the physician ultimately responsible. The Belgian Euthanasia Act (3) mandates consultation with the nursing team if they are involved in a patient's care with a pending euthanasia application. Nursing students may participate in preliminary decision-making. Exploring attitudes, skills, and roles, studies focussing on Belgian nursing students and graduated psychiatric nurses revealed a need for enhanced knowledge, skill development, and clear guidelines. Existing literature lacks specificity on euthanasia aspects related to terminal suffering or other reasons for requests (4-8).  In response to the expressed need of nursing students and graduated nurses for improved training in handling difficult and complex situations in euthanasia, a teaching module that utilizes simulation-based learning was developed as nursing students envision a clear role for themselves in the future but feel inadequately prepared for it. The focus extended beyond UMS-euthanasia as in previous studies (4-8), encompassing various reasons for requesting euthanasia because our aim is to equip nursing students with the necessary skills to engage in discussions about euthanasia in general with their patients. Simulation learning has proven to be an effective educational approach for training both students and registered nurses, with nursing interventions based on simulation showing a significant educational impact (8). | | |
| **METHODS** | | |
| Please provide more information about the Procedure and Tools section. For example, should all sessions be completed within a day, or are they divided into different days? In the Simulation Module section, should each group experience three scenarios, or does each group choose one? | | We thank the reviewer for these suggestions. |
| Please introduce data analysis in a separate section rather than combining it in the “Data Collection” section. | | We introduced “Data analysis” as a separate section. |
| The last part of the simulation module features three different simulation scenarios dealing with questions about life-end and euthanasia. Each simulation session included the enactment of all three scenarios. The presence of the perception of unbearable suffering is a defining characteristic of all scenarios.  An interview guide with open questions was used to explore the participants' perceptions on the simulation experience in general, and each particular phase of the simulation module (see S1 appendix).  The three simulation sessions and their respective discussion groups took place on separate days. | | |
| **RESULTS** | | |
| Please provide a table displaying the sociodemographic data of the participants. | | We have considered this matter but ultimately decided not to do so in order to ensure the anonymity of the participants, as there are older students among them who could potentially be identified with overly specific information, given that the involved programs are relatively small (n=134). |
| Suggest reporting the results following the typical structure of qualitative research, generalizing by theme and sub-theme, rather than by intervention implementation session. | | We have also considered this, but we have opted for this approach because it appears to be the most logical way to present our results. In this manner, we aim to maintain clarity and readability throughout the presentation of our findings, ensuring their comprehensibility to a broad audience. |
|  | | |
| **DISCUSSION** | | |
| Suggest moving strengths and limitations to the end of the Discussion and titling this section “Strengths and limitations”. | | We thank the reviewer for this suggestion. |
| Lines 419 to lines 420 mention the sample size, which consists of 17 students from two universities. It is unclear whether this is considered a strength of the study, clarification is needed. | | We clarified this in the “Strengths and limitations”. |
| Regarding the simulation module in itself, some strengths can be identified such as the participation of two University Colleges from two different Belgian provinces. By limiting the groups to six students, all participants were able to assume an active role during the simulation session, ensuring their active engagement rather than solely observatory participation. Additionally, there was not only a group from each respective institution but also a mixed group comprising students from both University Colleges. Besides the diversity of students, another strength is that the simulation module was validated by four external experts. | | |

| **REVIEWER 2** | |
| --- | --- |
| **ABSTRACT** | |
| The abstract lacks the research methods which were used to conduct the study. | We have revised the abstract based on your feedback. |
| The authors also did not write any recommendations yet they are important for readers to see the significance of the study. | We have revised the abstract based on your feedback. |
| Nursing students are confronted with euthanasia during their internship and certainly during their later career but they feel inadequately prepared in dealing with a euthanasia request. This study presents a simulation module focused on euthanasia and evaluates nursing students' perceptions after they have completed the simulation practice. The ‘euthanasia module’ consisted of a preparatory online learning module, a good-practice video, an in-vivo simulation scenario, and a debriefing session. The module’s content was validated by four experts in end-of-life care. The module was completed by three groups of students from two different University Colleges (n=17 in total). The students were divided into three groups: one with no previous simulation education experience, one with all students having previous experience, and another with a mix of experiences. After completing the entire module, each group had a discussion regarding their perceptions and expectations concerning this simulation module. Thematic content analysis was conducted on audio-recorded group interviews using NVIVO® software, involving initial open coding, transformation into specific themes and subthemes through axial coding, and defining core themes through selective coding, with data analysis continuing until data saturation was reached. Students generally found the online learning module valuable for background information, had mixed perceptions of the good-practice video, and appreciated the well-crafted scenarios with the taboo of euthanasia emerging during simulations, while the debriefing process was seen as enhancing clinical reasoning abilities. Students considered the simulation module a valuable addition to their education and nursing careers, expressing their satisfaction with the comprehensive coverage of the sensitive topic presented without sensationalism or taboos. This subject holds significance for nations with established euthanasia laws and those lacking such legal frameworks. The findings of this study can aid teachers in developing and accessing euthanasia simulation training programs, contributing to broader education's emphasis on integrating euthanasia-related knowledge and skills. | |
| **INTRODUCTION** | |
| The topics being studied was well introduce and the purpose of the study was described well. | We thank the reviewer for the appreciation of our work. |
| In lines 65 to 73, the authors cited a previous study and that description was too detailed. It was going to be better if this was a literature review, but since this an introduction, the authors should make their own argument and then support it with previous literature. | We thank the reviewer for its suggestion. We adapted the text. |
| The in-text referencing is wrongly done, It would never be before a sentence or after a full-stop. Please read about this. | We positioned all citations prior to the endpoint. |
| In Belgium, handling euthanasia requests follows a rigorous procedure, with the physician ultimately responsible. The Belgian Euthanasia Act (3) mandates consultation with the nursing team if they are involved in a patient's care with a pending euthanasia application. Nursing students may participate in preliminary decision-making. Exploring attitudes, skills, and roles, studies focussing on Belgian nursing students and graduated psychiatric nurses revealed a need for enhanced knowledge, skill development, and clear guidelines. Existing literature lacks specificity on euthanasia aspects related to terminal suffering or other reasons for requests (4-8). | |
| **METHODOLOGY** | |
| Methodology is this study was done poorly. Please discuss the methodology under these subtopics or at least make sure vital information from these aspects is not omitted in your article:  -Research setting  -Research design  -Study population and sampling  -Data collection  -Data analysis  -Ethical considerations | We have restructured the methods section to align with the proposed organization. |
|  | |
| **RESULTS** | |
| This is fine. | We thank the reviewer for the acceptation of this part of our paper. |
| **DISCUSSION** | |
| This is fine. | We thank the reviewer for the acceptation of this part of our paper. |
| **CONCLUSION** | |
| Your conclusion talks mostly on the significance of the study. Please write again about your results like you did on the abstract and the add recommendation. | We have revised the conclusion based on the suggestions. |
| This article describes the development of novel simulation module on how to deal with a euthanasia request. This work also describes students’ feedback as well as perceptions on it. This module is designed for nursing programs but could be envisaged for other professionals. The simulation module has added value to the students' education and future careers as nurses. Their positive feedback underscores the comprehensive coverage of this sensitive topic. The need for enhancing knowledge, skills, and clear guidelines in the domain of euthanasia-related education becomes apparent from the students' perceptions. The results of this study can support teachers in designing and evaluating similar simulation-based modules into their nursing programs, providing a structured and practical approach to addressing the complexities of euthanasia. It is important to tailor such modules to different geographical and legal contexts, ensuring their relevance and effectiveness in diverse settings. This research contributes to the broader educational landscape by emphasizing the integration of euthanasia-related knowledge and skills, ultimately better preparing nursing students for the challenges they may encounter in their future careers. | |
