# Supplementary material for: Using simulation to teach nursing students how to deal with a euthanasia request
Source: PLoS One. 2024 Mar 28;19(3):e0299049. doi: 10.1371/journal.pone.0299049 (PMC10977752; doi:10.1371/journal.pone.0299049)
Supplement: Attachment — Submitted filename: R2 Response to Reviewers.docx [file pone.0299049.s003.docx]

**REBUTTAL LETTER – RESPONSE TO THE EDITOR AND REVIEWERS**

**Manuscript ID PONE-D-23-21618**

**Using simulation to teach nursing students how to deal with a euthanasia request**

| **COMMENTS** | | **RESPONSE OF AUTHORS** |
| --- | --- | --- |
|  | | |
| **EDITOR** | | |
| The reviewers suggest some minor revisions to your manuscript. Therefore, I invite you to respond to the reviewer(s)' comments and revise your manuscript. | | We made the minor adjustments. These are legitimate comments. |
|  | |  |
| **REVIEWER 1** | | |
| The revised manuscript shows significant improvement, presenting a more coherent narrative. However, some minor points still require clarification. | We thank the reviewer for appreciating our previous adjustments. Further, we have tried to address both legitimate comments. | |
| The reference 4 to 8 are cited repeatedly in line 63. | We have rearranged the text to make it more readable. | |
| There is a separate paragraph just one sentence in line 84. Please make sure is it a mistake. | We have integrated this isolated sentence into the text. | |
| Exploring attitudes, skills, and roles, studies focussing on Belgian nursing students and graduated psychiatric nurses revealed a need for enhanced knowledge, skill development, and clear guidelines (4-8). In response to the expressed need of nursing students and graduated psychiatric nurses for improved training in handling difficult and complex situations in euthanasia, a teaching module that utilizes simulation-based learning was developed as nursing students envision a clear role for themselves in the future but feel inadequately prepared for it. The simulation session covers various reasons for requesting euthanasia because our aim is to equip nursing students with the necessary skills to engage in discussions about euthanasia in general with their patients.  To the best of our knowledge, no studies are currently available that report on the use of simulation based nursing education to prepare students for dealing with a euthanasia request, despite simulation's effectiveness. | | |

| **REVIEWER 2** | |
| --- | --- |
| The article is satisfactory. All the required specifications were met and the researchers did the suggested corrections. | We thank the reviewer for appreciating our previous adjustments. |
